# Construction of Built-In Electric Field in TiO_2_@Ti_2_O_3_ Core-Shell Heterojunctions toward Optimized Photocatalytic Performance

**DOI:** 10.3390/nano13142125

**Published:** 2023-07-21

**Authors:** Tingting Hu, Panpan Feng, Liping Guo, Hongqi Chu, Fusheng Liu

**Affiliations:** 1 State Key Laboratory Base for Eco-Chemical Engineering, College of Chemical Engineering, Qingdao University of Science and Technology, Qingdao 266042, China; hutingting_1981@163.com; 2 Shandong Provincial Key Laboratory of Molecular Engineering, School of Chemistry and Chemical Engineering, Qilu University of Technology (Shandong Academy of Sciences), Jinan 250353, China; guoliping@qlu.edu.cn (L.G.); hqchu@qlu.edu.cn (H.C.); 3 School of Chemistry and Pharmaceutical Engineering, Shandong First Medical University & Shandong Academy of Medical Sciences, Jinan 250117, China

**Keywords:** photocatalysis, Ti_2_O_3_@TiO_2_ core-shell heterojunction, built-in electric field

## Abstract

A series of Ti_2_O_3_@TiO_2_ core-shell heterojunction composite photocatalysts with different internal electric fields were synthesized using simple heat treatment methods. The synthesized Ti_2_O_3_@TiO_2_ core-shell heterojunction composites were characterized by means of SEM, XRD, PL, UV–Vis, BET, SPV, TEM and other related analytical techniques. Tetracycline (TC) was used as the degradation target to evaluate the photocatalytic performance of the synthesized Ti_2_O_3_@TiO_2_ core-shell heterojunction composites. The relevant test results show that the photocatalytic performance of the optimized materials has been significantly enhanced compared to Ti_2_O_3_, while the photocatalytic degradation rate has increased from 28% to 70.1%. After verification via several different testing and characterization techniques, the excellent catalytic performance is attributed to the efficient separation efficiency of the photogenerated charge carriers derived from the built-in electric field formed between Ti_2_O_3_ and TiO_2_. When the recombination of electrons and holes is occupied, more charges are generated to reach the surface of the photocatalyst, thereby improving the photocatalytic degradation efficiency. Thus, this work provides a universal strategy to enhance the photocatalytic performance of Ti_2_O_3_ by coupling it with TiO_2_ to build an internal electric field.

## 1. Introduction

Tetracycline (TC) has stable properties due to its four-complex benzene cycloalkyl groups [1,2]. It is highly susceptible to accumulation in the environment, and it is a difficult-to-degrade organic pollutant. Tetracycline (TC) in the environment is persistent, easy to accumulate, and difficult to degrade, which causes serious harm to the food chain and human health [3,4]. In addition, due to its long application, drug resistance is a serious concern. It can be absorbed from the gastrointestinal tract, albeit not completely. About 60%~70% of the dose is residual in the environment. Therefore, the long-term residue of TC in the aquatic environment harms human health and the ecological environment. To alleviate this serious situation, applying photocatalysis technology to the degradation of water pollutants as soon as possible will greatly help with the purification of the water environment [5,6]. Compared with traditional chemical, physical, and biological technologies, semiconductor photocatalytic technology can efficiently degrade organic pollutants in wastewater, such as antibiotic wastewater, into small-molecule substances [7,8,9].

As the ninth most abundant element on Earth, titanium has been widely studied for its high thermal stability, low cost, and light-responsive ability in metal oxide composites such as titanium dioxide (TiO_2_) [10,11,12]. TiO_2_ can be stimulated by light to produce strong oxidizing h^+^ and strong reducing e^−^, which makes TiO_2_ highly efficient in terms of its photocatalytic performance [13,14]. However, due to its inherent large bandgap (≈3 eV), typical titanium dioxide only responds to ultraviolet radiation with a wavelength of <378 nm [15,16,17]. In the past few decades, researchers have considerably reduced the bandgap to achieve titanium dioxide absorption of solar energy in the visible spectral range [18]. Research in the literature confirms that titanium dioxide’s bandgap width can be reduced to 1.5 eV, while its optical response can be extended to 800 nm, achieving a utilization rate of 40% for total solar energy [19]. However, to design an efficient full-spectrum solar converter with high solar energy utilization, further modification of titanium oxide is needed to reduce the bandgap to less than 0.5 eV.

Magnéli phase titanium oxides were only identified as conductive compounds in the 1950s, with the general formula Ti_n_O_2n−1_ (3 < *n* < 10). This non-stoichiometric semiconductor titanium oxide, which contains structural vacancies in both the titanium and oxygen sublattices, exhibits excellent photocatalytic performance. Due to the limitations of the methods used to manufacture Magnéli phase materials, it is difficult to prepare individual pure phases [20,21]. Therefore, the Magnéli phase materials synthesized by heating TiO_2_ or Ti at different temperatures typically contain mixed phases. In addition, the reliability of the electrical and chemical properties of the phases obtained through separation methods is relatively low. Even the most common methods (such as lame synthesis methods, both electrochemical and chemical) for synthesizing titanium suboxides still have many problems, such as the low active surface of the obtained material and the difficulty in adjusting the size and structure. For example, processing must be carried out in a high-temperature and oxygen-free atmosphere. In addition, even at temperatures ranging from 425 to 525 °C, Ti_n_O_2n−1_ is unstable and decomposes into various upper structures. However, the enhanced part of the photocatalytic performance with regard to photocatalytic degradation comes from the nanostructures on the surface of bulk titanium suboxides. Ti^3+^ ions play a significant role in reducing the bandgap and effectively boosting the adsorption and activation of reactants, which markedly enhances the light-responsive ability [22]. The relatively small bandgap of Ti_2_O_3_ enables it to absorb solar energy within the full spectral range [23,24]. In addition, Ti_2_O_3_ is one of the most representative examples of the Mott insulation system, and its metal–insulator transition characteristics are derived from the electronic correlation effect [25,26]. Due to its narrow bandgap, Ti_2_O_3_ exhibits high theoretical photocurrent density and high carrier mobility. After modification, the light absorption ability of traditional Ti_2_O_3_ can be further enhanced. Among many methods, constructing an internal electric field at the interface of the photocatalysts as a driving force for charge separation is considered an efficient method for achieving maximum carrier separation and driving target surface reactions in photocatalytic systems [27]. Specifically, photogenerated electrons will undergo reverse transfer driven by an electric field, greatly accelerating the separation of electron–hole pairs [28,29]. For example, Cui et al. constructed an internal electric field by preparing p–n homojunction perovskite solar cells, which directionally promoted the transport of photogenerated carriers, greatly reducing the carrier recombination losses and improving the power conversion efficiency [30].

As titanium suboxides are usually prepared via heat treatment under an inert or reductive atmosphere, there is a demand for methods to prepare nanostructured titanium suboxides via hydrothermal or other methods. During these synthesis processes, the phase structure of the material is not easily controlled, making it even more difficult to construct controllable heterojunctions in an orderly manner. Research has shown that introducing TiO_2_ (anatase)-based symbionts into the low oxidation state structure can significantly improve the conductivity of the composite and the properties related to photoluminescence [31]. Therefore, in this work, we combined the TiO_2_ and Ti_2_O_3_ to construct an orderly core-shell heterojunction, creating an internal electric field. In addition, the band structures of Ti_2_O_3_@TiO_2_ heterojunctions were investigated by means of UV–Vis spectroscopy, the Mott–Schottky curve, and surface photovoltage (SPV). Constructing an internal electric field has been proven to promote the transfer efficiency of photogenerated charges and improve photocatalytic activity. The correlation between built-in electric fields and photocatalytic performance was revealed based on multiple test characterizations. The possible enhanced photocatalytic degradation mechanism of tetracycline (TC) was proposed. This work provides a simple and universal method for regulating the built-in electric fields in photocatalysts that can be applied to environmental pollution purification.

## 2. Materials and Methods

### 2.1. Chemicals

The Ti_2_O_3_ chemicals and tetracycline (TC) were purchased from Aladdin. The ethanol and Nafion solution were purchased from Sigma–Aldrich. Deionized water was used in the experiments. The materials used were all of analytical grade (99%) and had not undergone purification treatment.

### 2.2. Methods

#### Synthesis of the Ti_2_O_3_@TiO_2_ Heterojunction

The Ti_2_O_3_@TiO_2_ heterojunction was synthesized via a simple heat treatment method. First, using commercial Ti_2_O_3_ as the research object, a Ti_2_O_3_@TiO_2_ heterojunction system was constructed in an orderly fashion using heat treatment methods. Then, 1 g of Ti_2_O_3_ was dispersed in a porcelain boat. Program heating was used to raise the temperature at 2 °C min^−1^ from room temperature to different predetermined temperatures. The temperature was kept constant for 2 h and cooled to room temperature. The reaction conditions for the preparation of the TiO_2_@Ti_2_O_3_ heterojunction were explored. Reaction temperatures of 100 °C, 200 °C, 300 °C, 400 °C, 500 °C, 550 °C, 600 °C, and 700 °C were selected to explore the photocatalytic activity of TiO_2_@Ti_2_O_3_ toward TC decomposition. The prepared catalysts were named Ti_2_O_3_-100, Ti_2_O_3_-200, Ti_2_O_3_-300, Ti_2_O_3_-400, Ti_2_O_3_-500, Ti_2_O_3_-550, Ti_2_O_3_-600, and Ti_2_O_3_-700, respectively.

### 2.3. Characterizations

The crystalline phase structures of the Ti_2_O_3_@TiO_2_ samples were determined via X-ray diffraction (XRD) (Rigaku, Tokyo, Japan) with Cu Kα irradiation. Field emission scanning electron microscopy (SEM) (HITACHI, SU8010, Tokyo, Japan) and transmission electron microscopy (TEM) (JEM-ARM200F, Tokyo, Japan) were performed to reveal the surface topography of the Ti_2_O_3_@TiO_2_ samples. The adsorption isotherm of the Ti_2_O_3_@TiO_2_ samples was used to measure the specific surface area, and a pore size distribution analyzer (BET, Micromeritics, ASAP2460, Norcross, GA, USA) with N_2_ as the adsorption medium was used in combination with analysis via the Brunauer–Emmett–Teller (BET) method. The carrier recombination was evaluated based on the photoluminescence (PL) spectra (Edinburgh, Livingston, FLS 980, Scotland, UK) with using a Xenon lamp (excitation wavelength 375 nm). UV–Vis diffuse reflectance spectroscopy was used to analyze the spectral response range of the Ti_2_O_3_@TiO_2_ (DRS, Shimadzu, SolidSpec-3700, Tokyo, Japan). The energy gap (Eg) of the Ti_2_O_3_@TiO_2_ and Ti_2_O_3_ was obtained via the Tauc plot method. Thermogravimetric analysis (TGA) was used to monitor the thermal stability performance of the Ti_2_O_3_@TiO_2_ and Ti_2_O_3_ on an SDT Q600 (TA Instruments, New Castle, DE, USA). The SPV transient was recorded using a tunable Nd:YAG laser (EKSPLA, NT 342/1/UVE) excited by pulses with a duration of 5 ns and wavelengths between 420 and 720 nm, and a sampling oscilloscope (GAGE, CS14200, sampling rate of 100 Mm/s) was used by applying logarithmic readings without averaging to avoid potential accuracy loss in a short period. An electrochemical station (CHI660E, Chenhua, Shanghai, China) with a conventional three-electrode system was employed to carry out the electrochemical measurements. The three-electrode system contained working, counter (carbon rod) and reference (Ag/AgCl) electrodes in 1 M KOH alkaline medium. The steps for making the working electrodes were as follows: 4 mg of catalysts was dispersed in 1 mL of mixed solution (water:ethanol = 3:1) before 10 μL was taken and added dropwise to the surface of 1 × 1 cm^−2^ ITO and then left to dry naturally.

### 2.4. Measurement of Photocatalytic Activity

The photocatalytic performances of the Ti_2_O_3_@TiO_2_ and Ti_2_O_3_ were mainly confirmed by examining the photocatalytic degradation under simulated visible light. First, 100 mg of catalyst was added to 100 mL of TC solution (5 mg/L). A mechanical stirrer was used to stir the solution with a speed range of 0–1500 rpm. Before irradiation, a 20 min dark reaction was performed to achieve adsorption–desorption equilibrium. Then, the reaction solution was irradiated using a 300 W Xenon lamp with AM 1.5 (the current was 1.8 A; PLS-SXE300D, Perfect Light, Beijing, China). During the photocatalytic reaction, 4 mL of the solution was withdrawn every 20 min and the concentration of TC was measured. Correspondingly, the absorbance of TC in the supernatant solution was measured at a detection wavelength of 357 nm using a UV–Vis spectrophotometer corresponding to the maximum adsorption for the solution. The photocatalytic activities of the Ti_2_O_3_@TiO_2_ and Ti_2_O_3_ were studied by analyzing the degradation curves of the tetracycline.

## 3. Results and Discussion

### 3.1. The Results of the TGA

**Figure 1:**
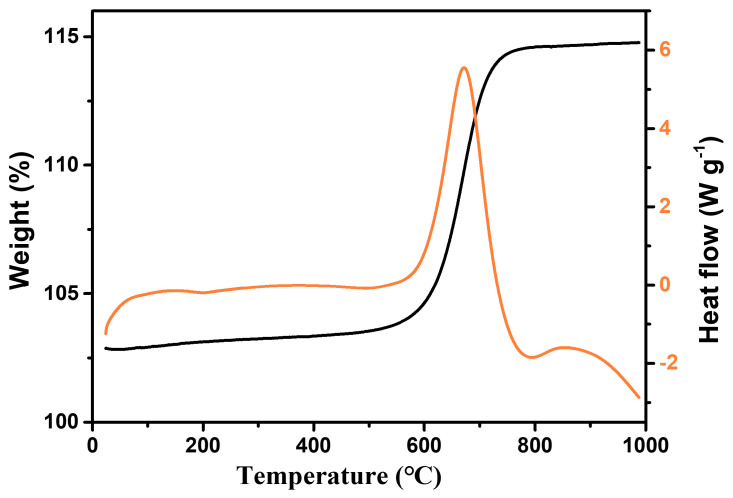
Optical photos of the Ti_2_O_3_@TiO_2_ heterojunction at different heat treatment temperatures.

To investigate the changes in the quality and heat flux of the materials at different temperatures, TG tests were conducted on the samples. From the results (Figure 1), it can be seen that when the temperature reaches above 500 °C, the material mass gradually increases, which is due to the gradual oxidation of Ti_2_O_3_ to form TiO_2_. When the temperature reaches 750 °C, the mass of the sample tends to stabilize. The sample undergoes phase transformation at different temperatures, so it is highly feasible to construct a Ti_2_O_3_@TiO_2_ heterojunction system in an orderly manner using heat treatment methods.

**Figure 2:**
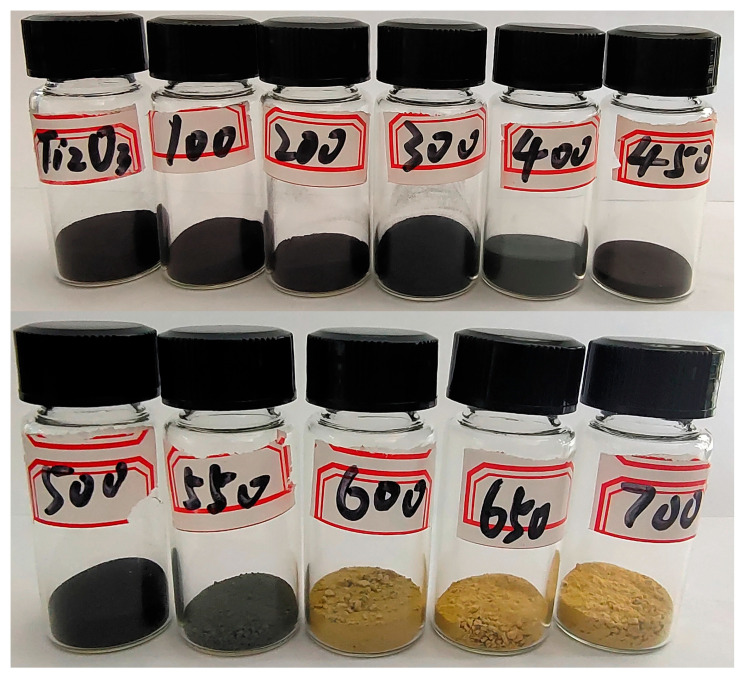
Optical photos of Ti_2_O_3_ at different heat treatment temperatures.

As shown in Figure 2, after treatment at different temperatures, the color of the Ti_2_O_3_ changed significantly, with the pure Ti_2_O_3_ turning black. When the temperature was below 600 °C, the color of the sample became lighter as the temperature increased. When the temperature was above 600 °C, the sample color turned yellow. Therefore, to further investigate the effect of temperature on the microstructures of the materials, scanning electron microscopy (SEM) was used to study the changes in the microstructure of the Ti_2_O_3_ under different heat treatment temperatures. Based on the results in Figure 1 and Figure 2, the Ti_2_O_3_, Ti_2_O_3_-400, Ti_2_O_3_-500, Ti_2_O_3_-550, Ti_2_O_3_-600, and Ti_2_O_3_-700 were selected for the SEM characterization.

### 3.2. The Results of the SEM and XRD Analyses 

**Figure 3:**
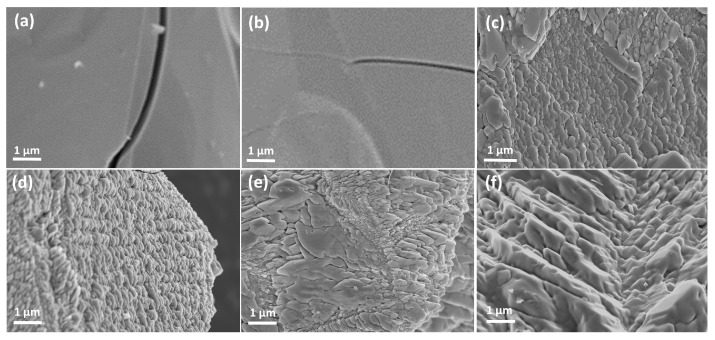
SEM images of Ti_2_O_3_ under different heat treatment temperatures: (**a**) Ti_2_O_3_; (**b**) Ti_2_O_3_-400; (**c**) Ti_2_O_3_-500; (**d**) Ti_2_O_3_-550; (**e**) Ti_2_O_3_-600; and (**f**) Ti_2_O_3_-700.

From the results in Figure 3, it can be seen that the Ti_2_O_3_ before heat treatment has an irregular 3D particle structure with a smooth surface. When the temperature is below 500 °C, there is no significant change in the microstructure and size of the sample. When the temperature reaches 550 °C, the surface roughness of the sample significantly increases and small particles are generated on the outer surface. As the temperature further increases, the outer particle diameter continuously increases and agglomeration occurs.

**Figure 4:**
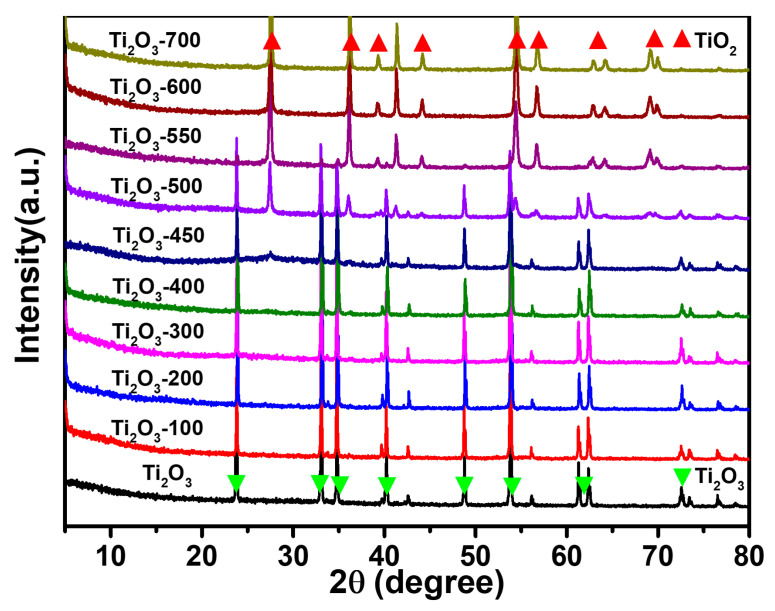
XRD spectra of Ti_2_O_3_, Ti_2_O_3_-100, Ti_2_O_3_-200, Ti_2_O_3_-300, Ti_2_O_3_-400, Ti_2_O_3_-450, Ti_2_O_3_-500, Ti_2_O_3_-550, Ti_2_O_3_-600, and Ti_2_O_3_-700.

In order to investigate the crystal form changes in Ti_2_O_3_ after the heat treatment reaction, the samples were characterized by measn of XRD. As shown in Figure 4, the diffraction peaks detected at 23.8°, 33.0°, 34.8°, 40.2°, 48.8°, and 53.7° are related to the Ti_2_O_3_ phase (JCPDS No. 43-1033) and can be attributed to the (012), (104), (110), (113), (024), and (116) crystal planes, respectively [32]. When the heat treatment temperature reaches 400 °C, new diffraction peaks begin to appear at 27.4° (110) and 36.0° (101), which belong to the rutile phase TiO_2_ (JCPDS No. 21-1276) [33]. With the further increase in temperature, the diffraction peaks attributed to the rutile phase TiO_2_ further appear at 27.4° (110), 36.0° (101), 39.2° (200), 41.2° (111), 44.0° (210), 54.3° (211), 56.6° (220), and 64.1° (310), and the peak intensity also increases with the thermal treatment temperature. When the temperature reaches 600 °C, all the Ti_2_O_3_ is converted into the TiO_2_ rutile phase. In addition, no diffraction peaks of impurities are observed. Therefore, Ti_2_O_3_ gradually undergoes phase transition into TiO_2_ during the heat treatment process. Combining XRD and SEM shows that the Ti_2_O_3_-450, Ti_2_O_3_-500 and Ti_2_O_3_-550 are composed of Ti_2_O_3_ and TiO_2_, forming a core-shell heterojunction. The experimental results show that the progress of TiO_2_ conversion can be controlled by changing the heat treatment temperature, thereby achieving the orderly construction of a Ti_2_O_3_@TiO_2_ heterojunction system.

### 3.3. The Results of the TEM Analyses 

**Figure 5:**
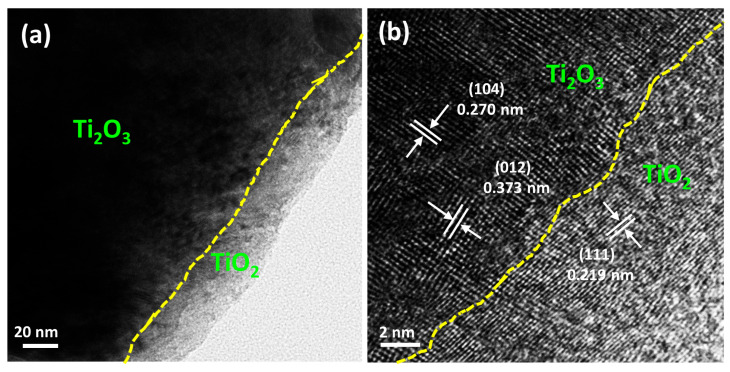
(**a**,**b**) High-resolution TEM image of the Ti_2_O_3_-550 after high-temperature heat treatment.

In order to further investigate the microstructure and crystal plane changes of the Ti_2_O_3_-based samples during the high-temperature reaction, we conducted TEM testing on the Ti_2_O_3_ samples after high-temperature treatment at 550 °C. Figure 5a shows that after the high-temperature treatment, there are significant differences between the microstructure of the outer layer and the internal structure of the Ti_2_O_3_@TiO_2_ heterostructure, such as the particle accumulation and size. As shown in Figure 5b, electron diffraction can further indicate that the particulate matter comprises Ti_2_O_3_ and TiO_2_. The high-resolution transmission test results show that the lattice fringes are d = 0.27 nm and 0.373 nm, belonging to the (104) and (0.12) of Ti_2_O_3_, and d = 0.219 nm, belonging to the (111) of TiO_2_. The TEM results are consistent with the previous XRD and scanning test results, once again proving that the heat treatment method can construct Ti_2_O_3_@TiO_2_ heterojunctions.

### 3.4. The Results of the N_2_ Adsorption Analyses 

**Figure 6:**
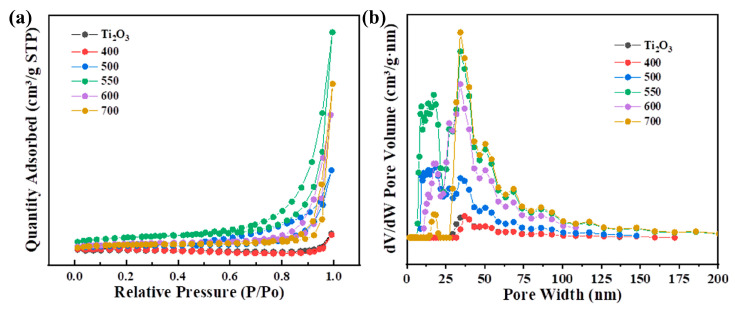
(**a**) N_2_ adsorption–desorption isotherms of Ti_2_O_3_ at different heat treatment temperatures. (**b**) Pore size distribution curve of Ti_2_O_3_ at different heat treatment temperatures.

N_2_ adsorption–desorption experiments were conducted to investigate the effects of different temperatures on the specific surface area and pore size of the composite materials. Figure 6a shows that the specific surface area of the Ti_2_O_3_-550 is the largest, approximately 0.796 m^2^/g, which is about 12 times that of the original Ti_2_O_3_ (approximately 0.067 m^2^/g). The pore volume of the Ti_2_O_3_ significantly increased from 0.000467 cm^3^/g to 0.0058 cm^3^/g (Ti_2_O_3_-550), demonstrating the reconstruction of the microstructure of the material during the transition from Ti_2_O_3_ to TiO_2_. In addition, in the Ti_2_O_3_@TiO_2_ heterojunction system, the Ti_2_O_3_-700 bulk phase has the highest content of TiO_2_, although its specific surface area is not the largest. This phenomenon proves that the expansion effect of the specific surface area in the system does not originate from the TiO_2_. Therefore, the research results indicate that the temperature has significant control over the microstructure of the system. In addition, from the pore size distribution map (Figure 6b), it can be seen that the pore size of the original Ti_2_O_3_ is mainly distributed around 37.5 nm and 50 nm. With the increase in the heat treatment temperature, the Ti_2_O_3_@TiO_2_ heterojunctions generate new pore structures in the bulk phase after retaining the original pore structure of the material, with the pore sizes mainly being concentrated in the range of 18–20 nm. The results suggest that the microstructure of the Ti_2_O_3_ did not undergo significant changes during the heat treatment process.

### 3.5. The Results of the UV–Vis Analyses 

**Figure 7:**
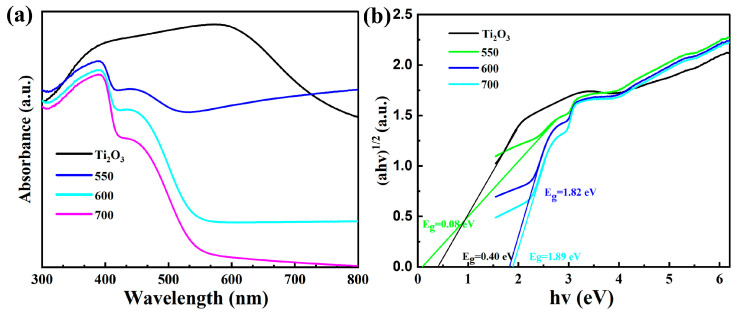
(**a**) Ultraviolet visible absorption spectra of the Ti_2_O_3_ and Ti_2_O_3_/TiO_2_ heterojunctions (Ti_2_O_3_-550, Ti_2_O_3_-600, and Ti_2_O_3_-700) and (**b**) the corresponding Kubelka–Munk conversion reflection spectra.

UV visible absorption spectroscopy was used to study the regulatory effect of the built-in electric field on the optical properties of the Ti_2_O_3_ and Ti_2_O_3_@TiO_2_ heterojunctions. As shown in Figure 7, both the Ti_2_O_3_ and Ti_2_O_3_@TiO_2_ heterojunctions exhibit strong absorption from 200 to 500 nm. It is worth noting that the Ti_2_O_3_-550 sample exhibits excellent absorption capacity throughout the entire process and significant tail absorption under the irradiation of visible light (λ > 600 nm). Compared with the as-received Ti_2_O_3_, the absorption ability of the Ti_2_O_3_-550 sample in the visible light absorption region remains unchanged, or even slightly enhanced. The existence of an internal electric field can promote the absorption ability of the Ti_2_O_3_-550 with regard to visible light. In addition, the bandgaps of the Ti_2_O_3_ and Ti_2_O_3_@TiO_2_ heterojunctions can be estimated using the formula of αhv=A(hv−Eg)12 (Eg is the bandgap energy, α is the absorption coefficient, A is a constant, v is the optical frequency, and h is the Planck constant) [34]. In the graph of photon energy, the bandgap energy value is the intersection point between the extended dashed line and the horizontal axis of the coordinate system (the horizontal axis value is hv = 1240/wavelength, while the vertical axis value is (α Hv) ½) [35,36]. As shown in Figure 7b, the calculated bandgaps of the pure Ti_2_O_3_ and Ti_2_O_3_-550, Ti_2_O_3_-600, and Ti_2_O_3_-700 are 0.4 eV, 0.08 eV, 1.82 eV, and 1.89 eV, respectively, indicating that the bandgaps of semiconductor materials can be effectively adjusted and optimized by constructing a reasonable internal electric field. Compared with other reported materials, the synthesized materials have relatively small bandgaps (Table 1).

### 3.6. The Results of the Optoelectronic Performance Analyses 

**Figure 8:**
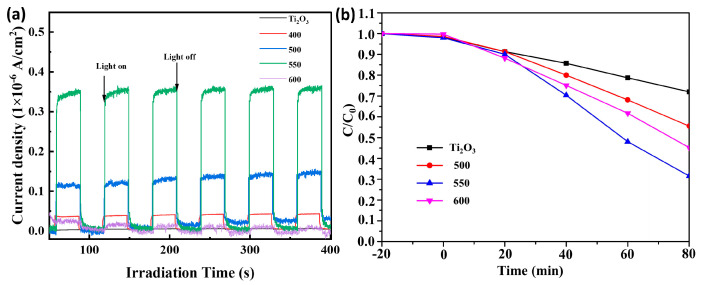
(**a**) Photogenerated current of the pure Ti_2_O_3_ and Ti_2_O_3_-400, Ti_2_O_3_-500, Ti_2_O_3_-550, and Ti_2_O_3_-600, and (**b**) the photocatalytic degradation performance of tetracycline.

Photoelectrochemical measurements in a typical three-electrode battery were used to study the electron generation and migration characteristics of the Ti_2_O_3_ and Ti_2_O_3_@TiO_2_ heterojunctions. As shown in Figure 8a, among the pure Ti_2_O_3_ and Ti_2_O_3_@TiO_2_ heterojunction systems, the Ti_2_O_3_-550 has the strongest photocurrent density, much higher than the original Ti_2_O_3_, confirming its excellent solar energy utilization ability. The above measurements intuitively prove that the material prepared at 550 °C has the best photoelectric performance. In addition, the relationship between the samples prepared at different heat treatment temperatures and the photocatalytic performance was systematically studied. The catalytic performance of the Ti_2_O_3_ and Ti_2_O_3_@TiO_2_ heterojunctions (Ti_2_O_3_-400, Ti_2_O_3_-500, Ti_2_O_3_-550, Ti_2_O_3_-600) under visible light was investigated using TC as the substrate. As shown in Figure 8b, after stirring under dark conditions for 20 min, the matrix adsorption–desorption equilibrium of all the samples was observed. The photocatalytic performance of the Ti_2_O_3_ and Ti_2_O_3_@TiO_2_ heterojunction composite materials was evaluated by measuring the degradation of the tetracycline in the sample under simulated visible light irradiation. From the results, it can be seen that Ti_2_O_3_ significantly enhances its ability to degrade tetracycline by constructing a Ti_2_O_3_@TiO_2_ heterojunction, with the degradation rate increasing from 28% to 70.1% (35.05 mg of tetracycline degraded/100 mg of photocatalyst). Compared with other reported materials, the synthesized materials have relatively good photocatalytic degradation efficiency (Table 2). As the temperature further increases, the catalytic efficiency decreases. The experimental results indicate that the excellent photocatalytic performance of the Ti_2_O_3_-550 can be attributed to the narrower bandgap, reasonable band structure, and larger specific surface area. More importantly, the proper construction of the heterojunction may result in the redistribution of the electron density to build an internal electric field in the Ti_2_O_3_@TiO_2_ and inhibit the recombination process of the electron–hole pairs. Therefore, by constructing heterojunctions reasonably and achieving precise regulation of the built-in electric fields, the photocatalytic activity of semiconductor materials can be optimized.

### 3.7. Discussion on the Photocatalytic Mechanism

**Figure 9:**
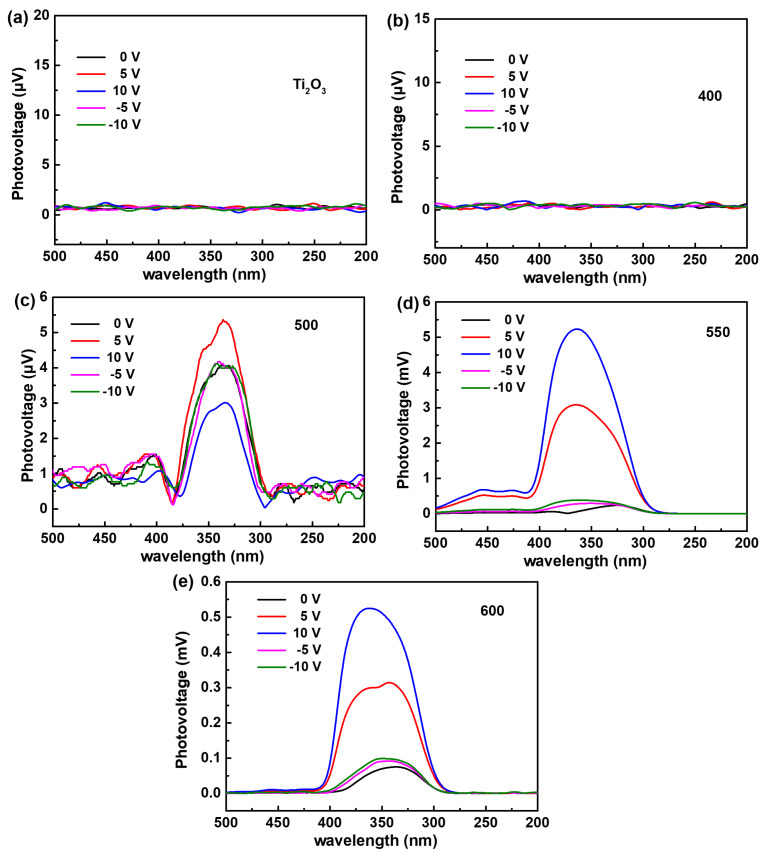
Surface photovoltage (SPV) diagrams of the Ti_2_O_3_ and Ti_2_O_3_/TiO_2_ heterojunctions: (**a**) Ti_2_O_3_, (**b**) Ti_2_O_3_-400, (**c**) Ti_2_O_3_-500, (**d**) Ti_2_O_3_-550, and (**e**) Ti_2_O_3_-600.

In order to investigate the built-in electric field effect in the Ti_2_O_3_ and Ti_2_O_3_@TiO_2_ heterojunctions in depth, the surface photovoltage (SPV) was used to investigate the separation and migration process of the photogenerated charge carriers in the materials [48]. Figure 9a,b show that the SPV response signals of the original Ti_2_O_3_ and Ti_2_O_3_-400 were not detected. This may be due to the materials’ low separation efficiency or the high recombination efficiency of the photogenerated carriers. When the heat treatment temperature was further increased to 500 and 550 °C (Figure 9c,d), significant photovoltage response signals were observed at 275–400 nm for the Ti_2_O_3_-500 and Ti_2_O_3_-550, which can be attributed to the electronic transitions between the valence band and conduction band [49,50]. Moreover, the SPV response is positive, indicating that the upward energy band bends toward the surface/interface [51]. It can be clearly seen that the SPV response values have increased to 4.1 μV and 2.33 mV, respectively. This increase is caused by the built-in electric field constructed by the Ti_2_O_3_ and TiO_2_, which continuously adjusts with the composition. With the increase in the heat treatment temperature, the amount of TiO_2_ in the outer layer from the conversion of the Ti_2_O_3_ gradually increases and the built-in electric field effect is continuously enhanced. Thus, the separation of the photoexcited charge carriers is promoted, resulting in changes in the surface photovoltage. When the direction of the applied electric field is changed, there is no significant change in the photogenerated voltage of the Ti_2_O_3_-500 (Figure 9c), indicating that the direction of the built-in electric field of the Ti_2_O_3_-500 is opposite to the direction of the positive applied electric field. As shown in Figure 9d, the photogenerated voltage of the Ti_2_O_3_-550 significantly increases with the applied electric field. When the direction of the applied electric field is changed, the photogenerated voltage of the Ti_2_O_3_-500 is suppressed and reduced, indicating that the direction of the built-in electric field of the Ti_2_O_3_-550 is the same as the direction of the positive applied electric field. However, when the heat treatment temperature is increased to 600 °C (Figure 9e), the outer TiO_2_ content further increases. The SPV response of the Ti_2_O_3_-600 decreases to 0.75 mV, indicating that in the presence of excessive TiO_2_, the built-in electric field effect decreases and the separation of the photoexcited charge carriers and the recombination of the photogenerated electron–hole pairs are suppressed. In addition, the photogenerated voltage of the Ti_2_O_3_-600 increases significantly with the increase in the applied electric field. When the direction of the applied electric field changes, the photogenerated voltage of the Ti_2_O_3_-600 is suppressed and reduced, indicating that the direction of the built-in electric field of the Ti_2_O_3_-600 is the same as that of the positive applied electric field. Photogenerated charge carriers can recombine through radiation or non-radiation decay. Therefore, by comparing the changes in the SPV response with the heat treatment temperature in the Ti_2_O_3_ and Ti_2_O_3_@TiO_2_ heterojunctions, it can be inferred that the Ti_2_O_3_-550 has the best built-in electric field effect, which can effectively suppress non-radiative decay, allowing for the more effective separation of the photogenerated charge carriers and transport of the photogenerated electrons to the catalyst surface to facilitate the degradation of the tetracycline [52,53].

**Figure 10:**
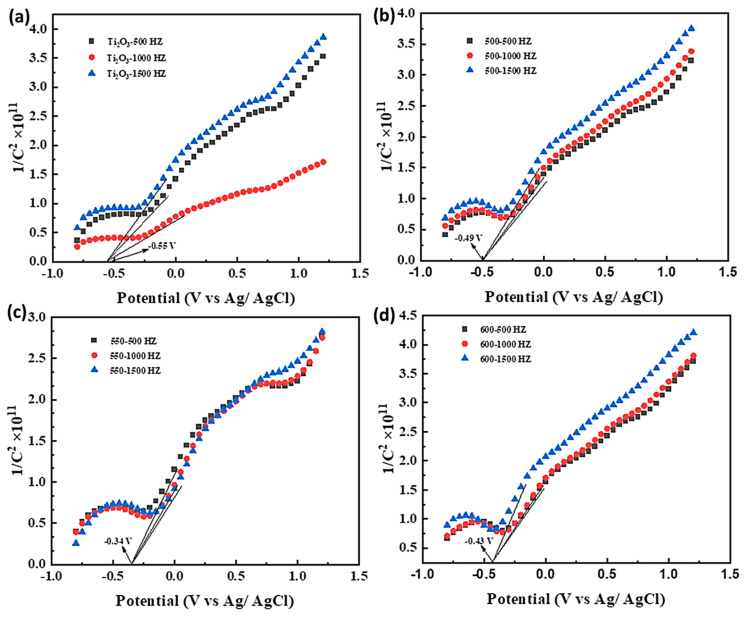
Mott–Schottky diagrams of the Ti_2_O_3_ and Ti_2_O_3_/TiO_2_ heterojunctions: (**a**) Ti_2_O_3_, (**b**) Ti_2_O_3_-500, (**c**) Ti_2_O_3_-550, and (**d**) Ti_2_O_3_-600.

As shown in Figure 10, the slope of the straight-line portion of the Mott–Schottky curve of the Ti_2_O_3_ and Ti_2_O_3_@TiO_2_ heterojunctions (Ti_2_O_3_-500, Ti_2_O_3_-550, and Ti_2_O_3_-600) within the passivation zone is positive, indicating that the semiconductor film formed by the composite materials in this solution is an n-type semiconductor [54,55]. When the Ti_2_O_3_@TiO_2_ heterojunction is not in contact with the solution, the material’s Fermi energy level is higher than the solution’s chemical potential. When the Ti_2_O_3_@TiO_2_ heterojunction enters the solution, the carriers, i.e., electrons, in the bulk phase will spontaneously transfer from the high energy level to the low energy level, that is, from the side of the Ti_2_O_3_@TiO_2_ to the solution, to balance the Fermi energy levels [56]. A space charge layer is formed on one side of the Ti_2_O_3_@TiO_2_ film, causing the bulk phase to carry opposite charges to the solution. Excess charges are distributed within the space charge layer. Due to the lower concentration of electrons in the Ti_2_O_3_@TiO_2_ bulk phase compared to the solution, electrons continue to enter the solution. Therefore, in the surface area on the semiconductor side, electrons are consumed, leaving only positive charges. This process causes the energy ratio near the surface to be corrected internally in the semiconductor, resulting in the energy bands of the Ti_2_O_3_ and Ti_2_O_3_@TiO_2_ heterojunctions bending upwards in this region. As electrons continue to propagate, the energy band continues to bend upwards. If an external voltage is continuously applied to the Ti_2_O_3_ and Ti_2_O_3_@TiO_2_ heterojunctions, the charge distribution at the interface changes with the electron input and the band bending changes. When the electrons that flood into the space charge layer neutralize the excess positive charge there, the energy band of the semiconductor returns to the same level as when it was not in contact with the solution. At this point, the required voltage is the flat band voltage. Suppose there is a built-in electric field in the semiconductor. In that case, it will decrease the necessary external voltage required to determine the potential size of the built-in electric field.

As shown by the results, the flat band potentials of the Ti_2_O_3_, Ti_2_O_3_-500, Ti_2_O_3_-550, and Ti_2_O_3_-600 are −0.55 V, −0.49 V, −0.34 V, and −0.43 V, respectively. The results indirectly prove that in the Ti_2_O_3_@TiO_2_ heterojunction system, the built-in electric field of the Ti_2_O_3_-550 has the highest potential. In addition, the results of the Mott–Schottky curve indicate that the Ti_2_O_3_-550 has the highest carrier concentration, which is consistent with the conclusion obtained via the SPV. The Ti_2_O_3_-550 has a high Fermi energy level, which can endow the Ti_2_O_3_@TiO_2_ heterostructure with a more excellent electronic reservoir capability and accelerate the carrier separation and conversion.

**Figure 11:**
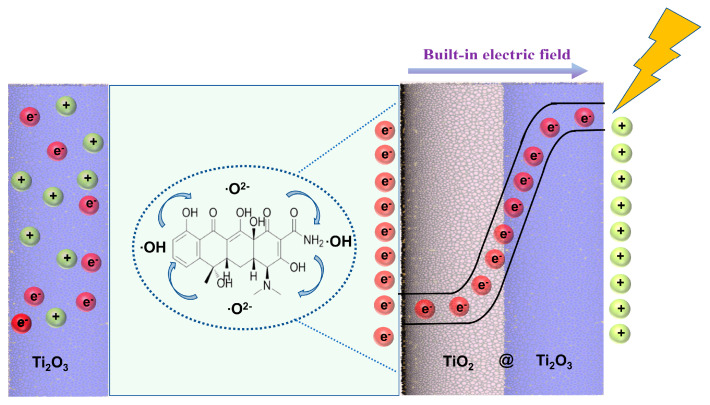
Schematic diagram of photocatalytic TC degradation over the Ti_2_O_3_@TiO_2_ heterojunctions.

These experimental results indicate that under visible light irradiation, the photocatalytic reaction of the Ti_2_O_3_@TiO_2_ heterojunction is direct photocatalysis, where electrons are excited by visible light from the valence band of the TiO_2_ to the transition band of the Ti_2_O_3_, which is lower than the conduction band of the TiO_2_. Then, electrons can easily transfer from the Ti (III) site to the adjacent Ti (IV) sites through the valence excitation process [22]. Under visible light irradiation, photogenerated holes are injected into the conduction band of the photocatalyst by the built-in electric field. Subsequently, electrons are captured to produce reactive substances and induce degradation reactions (Figure 11).

## 4. Conclusions

In summary, Ti_2_O_3_@TiO_2_ heterojunctions have been constructed via a sample heat treatment method as an efficient TC degradation system. The photocatalytic activity of the Ti_2_O_3_@TiO_2_ heterojunctions for TC degradation under visible light was investigated. Compared with the pure Ti_2_O_3_, the photocatalytic performance of the Ti_2_O_3_@TiO_2_ heterojunctions was significantly improved. After the heterojunction structure was constructed, the photodegradation rate increased from 28% to 70.1% (35.05 mg of tetracycline degraded/100 mg of photocatalyst). The relevant experimental results indicated that the excellent photocatalytic performance of the Ti_2_O_3_-550 can be attributed to the narrow bandgap and reasonable band structure derived from the built-in electric field. By using the SPV and Mott–Schottky methods, it was revealed that the construction of the heterojunctions in the Ti_2_O_3_@TiO_2_ resulted in the construction of an internal electric field, which suppressed the recombination process of the photogenerated electron–hole pairs. The most significant aspect here is that the built-in electric field can be controlled by changing the content of Ti_2_O_3_ and TiO_2_. Overall, this work provides a simple method to precisely regulate the built-in electric field and optimize the photocatalytic activity of semiconductor materials by reasonably constructing heterojunctions.

## Figures and Tables

**Table 1:** Comparison of the bandgaps of Ti_2_O_3_@TiO_2_ and other reported studies.

Number	Titanium-Based Oxides	Bandgap	References
1	α-Ti_2_O_3_	1–2 eV	[37]
2	csp-Ti_2_O_3_	1–2 eV	[37]
3	Anatase TiO_2_	3.2 eV	[38]
4	TiO_2_	3.3 eV	[39]
5	α-Ti_2_O_3_	≈0.1 eV	[23]
6	TiO_2_	3.23 eV	[40]
7	Ti_2_O_3_	3.18 eV	[40]
8	T-723 (TiO_x_@anatase)	3.04 eV	[41]
9	T-810 (TiO_x_@anatase)	3.1 eV	[41]
10	Ti_2_O_3_@TiO_2_	0.08 eV	This work

**Table 2:** Comparison of the degradation efficiency of Ti_2_O_3_@TiO_2_ and other reported studies.

Number	Titanium-Based Oxides	Degradation Efficiency (mg of Tetracycline Degraded/mg of Photocatalyst)	References
1	TiO_2_	5.67/20	[1]
2	MnTiO_3_/Ag/gC_3_N_4_	6.1/10	[42]
3	TBM0.05–5	0.66/10	[8]
4	Ag/ZnO@BC	0.703/10	[43]
5	Bi_3_O4Br	0.622/20	[44]
6	CTF-Bi_2_WO_6_	0.771/20	[45]
7	2.0%Au/BiOCOOH	1.13/20	[46]
8	g-C_3_N_4−x_/g-C_3_N_4_	1.56/50	[47]
9	Pristine TiO_2_	0.63/10	[8]
10	Ti_2_O_3_@TiO_2_	35.05/100	This work

## Data Availability

Not applicable.
